# Monitoring Weeder Robots and Anticipating Their Functioning by Using Advanced Topological Data Analysis

**DOI:** 10.3389/frai.2021.761123

**Published:** 2021-12-13

**Authors:** Tarek Frahi, Abel Sancarlos, Mathieu Galle, Xavier Beaulieu, Anne Chambard, Antonio Falco, Elias Cueto, Francisco Chinesta

**Affiliations:** ^1^ PIMM Lab and ESI Group Chair, Arts et Metiers Institute of Technology, Paris, France; ^2^ ESI Group, Rungis, France; ^3^ VITIROVER, Saint-Emilion, France; ^4^ ESI-CEU International Chair CEU-UCH, Departamento de Matematicas, Fisica y Ciencias Tecnologicas, Universidad Cardenal Herrera-CEU, Valencia, Spain; ^5^ Aragon Institute of Engineering Research, Universidad de Zaragoza, Zaragoza, Spain

**Keywords:** autonomous robots, monitoring, topological data analysis, trajectory analysis, artificial intelligence, data classification

## Abstract

The present paper aims at analyzing the topological content of the complex trajectories that weeder-autonomous robots follow in operation. We will prove that the topological descriptors of these trajectories are affected by the robot environment as well as by the robot state, with respect to maintenance operations. Most of existing methodologies enabling efficient diagnosis are based on the data analysis, and in particular on some statistical quantities derived from the data. The present work explores the use of an original approach that instead of analyzing quantities derived from the data, analyzes the “shape” of the data, that is, the time series topology based on the homology persistence. We will prove that this procedure is able to extract valuable patterns able to discriminate the trajectories that the robot follows depending on the particular patch in which it operates, as well as to differentiate the robot behavior before and after undergoing a maintenance operation. Even if it is a preliminary work, and it does not pretend to compare its performances with respect to other existing technologies, this work opens new perspectives in considering quite natural and simple descriptors based on the intrinsic information that data contains, with the aim of performing efficient diagnosis and prognosis.

## 1 Introduction

Autonomous robots follow a number of rules introduced into their controllers (Alatise and Hancke, 2020; Shalal et al., 2013; Mohanty and Parhi, 2013). However, when they interact with the environment, small variations may result in long-time unpredictable motion. This behaviour is very usual in mechanics, characterizing systems exhibiting deterministic chaos (Avanço et al., 2016; Gupta et al., 2014).

**FIGURE 1:**
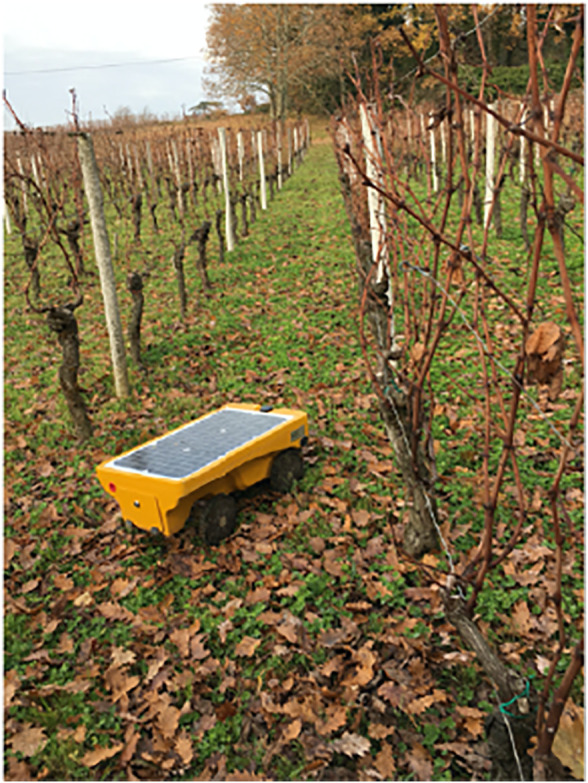
Weeder robot from VITIROVER micro robotique viticole.

In the practical case addressed in the present paper, a weeder robot (usually a float of them) is expected to cover a patch of a vineyard, in an optimal manner. Here, “optimal manner” refers to the path-line that allows covering the whole patch in a minimum time. However, the ground orography has a significant variability, as well as the location of the grapes. Robots are aimed at colliding the grape foots in order to remove the grass around, and then numerous collisions following different directions are needed to ensure that all the grass around the grape foot is adequately removed. Figure 1 depicts a VITIROVER MOWER ROBOT (https://www.vitirover.fr/en-robot for the technical specifications) considered in the present study under operational conditions.

The environmental variability (ground, grape location, grass distribution and size, and fixed and mobile obstacles) as well the intrinsic sensibility of the dynamics to small perturbations in the physical and operational conditions, provides an uncertain environment that makes useless the use of a deterministic framework for anticipating the robot trajectory. Thus, a random motion framework seems to be the most useful alternative.

In practice, to avoid under-performances characteristic of fully random motion, random motion operating at the local scale is combined with a more global deterministic planning that tries to better control the vineyard coverage by sequencing the operation at the different local patches covering the whole domain (Kavraki et al., 1998).

The present work does not aim at addressing such optimized operation conditions that will be addressed in a future publication under progress, but it aims at analyzing the data collected from a robot operating in different patches and under different conditions (with respect to the maintenance operations) in order to identify the existence of patterns able to identify the particular patch in which the robot operates, or to distinguish the different robot states with respect to the maintenance operations.

Having a sort of QR-code or identity card of each robot, when it operates within each patch, in a particular state (healthy or unhealthy), is of major relevance with respect to the predictive or operational maintenance of robots or floats of autonomous robots (Kavraki et al., 1998).

Most of existing methodologies enabling efficient diagnosis are based on the data analysis, and in particular on some statistical quantities derived from the data (Lhermitte et al., 2011) while the present paper aims at extracting information to be transformed into knowledge, from the data collected from each weeder robot, in particular the positions visited by the robot during its operation, and more concretely the topology contained in this data. Our goal is to extract the maximum information that could serve for differentiating them, enabling unsupervised clustering and/or supervised classification, prior to any action concerning diagnosis or modeling based on the use of adapted regressions. This first work aims at introducing a new methodology and does not pretend to compare its performances with respect to other existing technologies, comparison that will be addressed in a future work.

## 2 Methods

Using data clustering is almost straightforward, as soon as data is homogeneous and quantitatively expressible using integer or real numbers, enabling boolean or algebraic operations (addition, multiplication, … ). The interest of organizing data in groups, in a supervised or unsupervised manner, is that it is assumed that data belonging to a given group shares some qualities with the members of the group (Hastie et al., 2009; Murphy, 2012).

When proceeding in an unsupervised manner, the only information to group the data consists of the distance among them. Data that remain close to each other are expected to share some properties or behavior. This is the rationale considered in the very popular *k* *-means* technique (MacQueen, 1967; MacKay, 2003). However, the notion of proximity, leading to the derived concept of similarity, needs for the definition of a metric for comparison purposes. When data are well defined in a vector space, distances can be defined and data can be compared accordingly. In the case of supervised classification one is looking for the linear (or non-linear) Frontier separating the different groups on the basis of a quality or property that drives the data clustering. In this last case, the best Frontier separating two groups of data is the one maximizing the distance of the available data to the Frontier, in order to maximize the separation robustness. This is how support vector machine, SVM, works, for instance (Cristianini and Shawe-Taylor, 2000).

In both cases (supervised and unsupervised) the existence of a metric enabling data comparison is assumed. However, very often data could be much more complex, as for example when it concerns heterogeneous information, possibly categorial or qualitative. This is for example the case when a manufactured part is described by its identity card consisting of the name of the employee involved in the operation, the designation of the employed materials (some of them given by its commercial name), the temperature of the oven in which the part was cured and the processing time. In that case, comparing two parts becomes quite controversial if the employed metric is not properly defined. In these circumstances, usually, metrics are learned from the existing training data, as is the case when using decision trees (or its random forest counterpart) (Kirkwood, 2022; Breiman, 2001), code-to-vector Martín et al. (2019) or neural networks Goodfellow et al. (2016).

The situation becomes even more extreme when data have a large and deep topology content. This is the case for example of time series or images of rich microstructures. These are usually encountered in material science when describing metamaterials (also called functional materials), or those exhibiting gradient of properties or mesoscopic architectures. Thus, even in nominal conditions, time series will differ if they are compared from their respective values at each time instant. That is, two time series, even when they describe the same system in similar conditions, never match perfectly. Thus, they differ even if they resemble in a certain metric that should be learned. For example, our electrocardiogram measured during two consecutive minutes will exhibit a resemblance, but certainly both of them are not identical, thus making a perfect match impossible. A small variation will create a misalignment needing for metrics less sensible to these effects. The same rationale applies when comparing two profiles of a rough surface, two images of a foam taken in two close locations, … they exhibit a resemblance even if they do not perfectly match.

Thus, techniques aiming at aligning data were proposed. In the case of time-series, Dynamic Time Warping, DTW (Müller, 2007; Senin, 2008) has been successfully applied in many domains. The theory of optimal transport arose as a response to similar issues (Villani, 2006).

Another route consists of renouncing to *align* the data, and focussing on extracting the adequate, goal-oriented descriptors of these complex data, enabling comparison, clustering, classification and modelling (from non-linear regressions) (Lhermitte et al., 2011).

A first possibility consists of extracting the main statistical descriptors of time series or images (moments, correlations, covariograms, … ) (Torquato, 2002). Sometimes, data expressed in the usual space and time domains, are transformed into other spaces where their manipulation is expected to be simpler, like Fourier, Laplace, DCT, Wavelet, … descriptions of data. The most valuable (in the sense given later) descriptions seem to be those maximizing sparsity. These are widely considered when using compressed sensing (Ibañez et al., 2019), because it represents a compact, concise and complete way of representing data that seemed much more complex in the usual physical space (space and time).

The present work considers this last route, but uses a description based on the topology of data, described later, and successfully considered in our former works for addressing complex mesostructures Yun et al. (2020), time-series Frahi et al. (2021a), rough surfaces Frahi et al. (2020) and shapes Frahi et al. (2021b), with the aim of classifying and also constructing robust regressions expressing properties or performance from the input data expressed from its topological description.

The present study, when compared with our former developments, addresses a new and complex purpose: how the topology contained in the trajectory that an autonomous robot follows in a cloudy environment (where interactions limits the predictability horizon) can inform on the robot location (which patch into the whole vineyard) or the robot state (with respect to maintenance operations).

### 2.1 Data Description

In the study that follows, we consider a dataset consisting of the *x* and *y*-coordinates, calculated from the GPS longitudes and latitudes, representing the recorded position of the robot at time *t*:

$$D=\left\{\left(x\left(t\right),y\left(t\right),t\right),\;t\in T\right\}.$$

**FIGURE 2:**
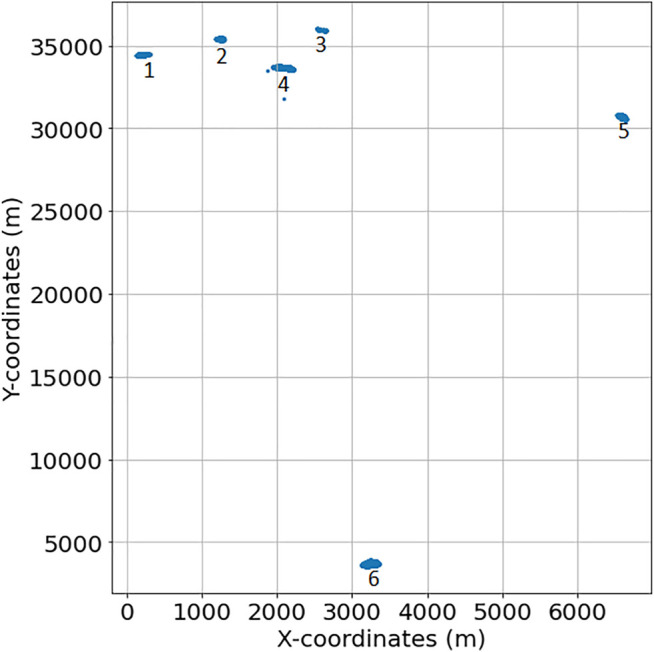
Location of the different patches.

**FIGURE 3:**
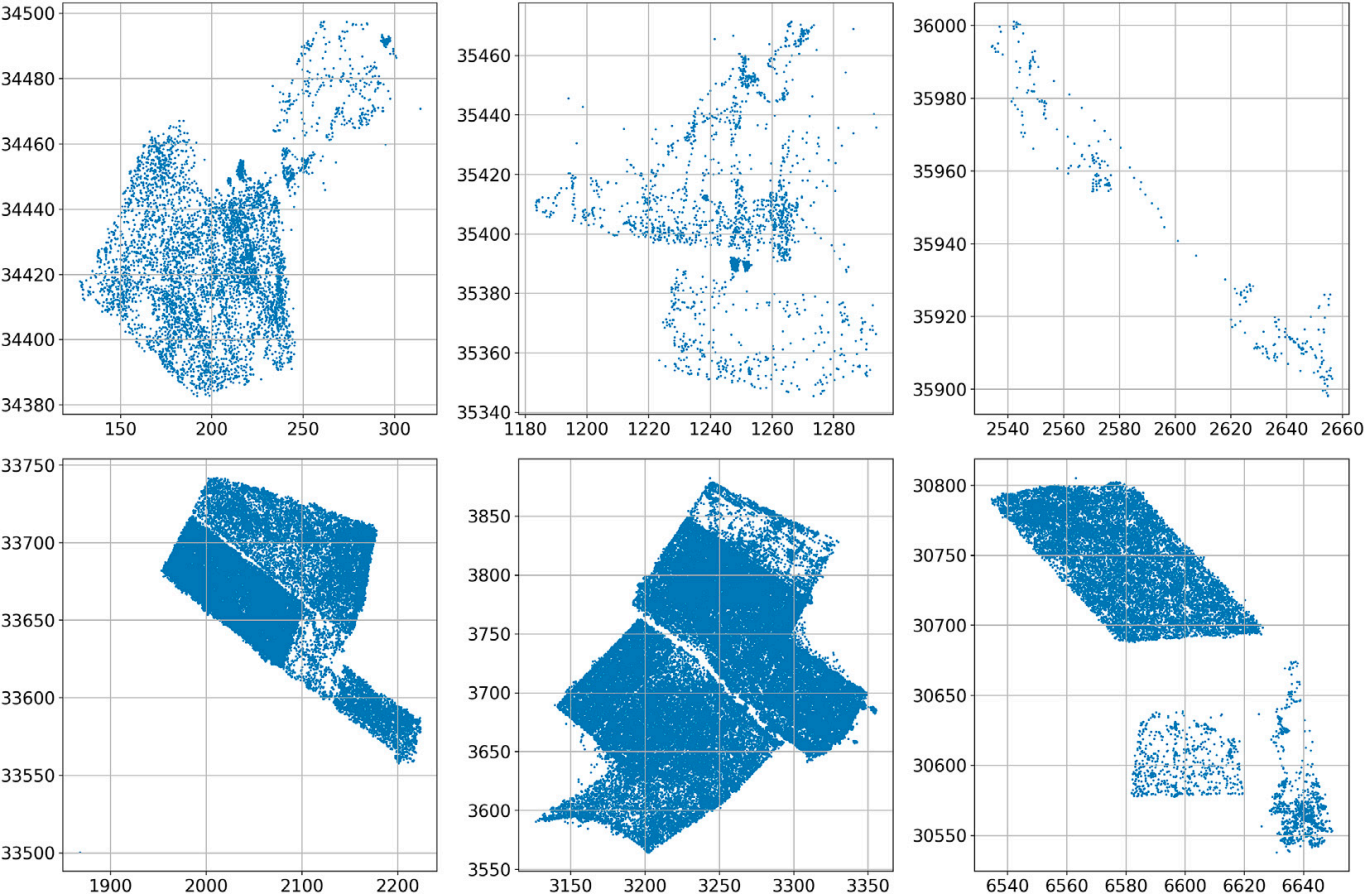
Robot trajectories in the six considered vineyard patches, from right to left and top to bottom: 1, 2, 3, 4, 5 and 6 (units in meters).

These coordinates span six different disjoint geographical patches within the whole vineyard, as illustrated in Figure 2, that have been recorded in a period of time T leading to the maps reported in Figure 3 that reflects the robot’s trajectory.

Maintenance operations are also known and properly identified in the provided dataset. Thus, the dataset consists of a collection of *n* discrete, finite and compact two-dimensional trajectories $S_{1},\ldots ,S_{n}$ .

### 2.2 Geometrical Features

**FIGURE 4:**
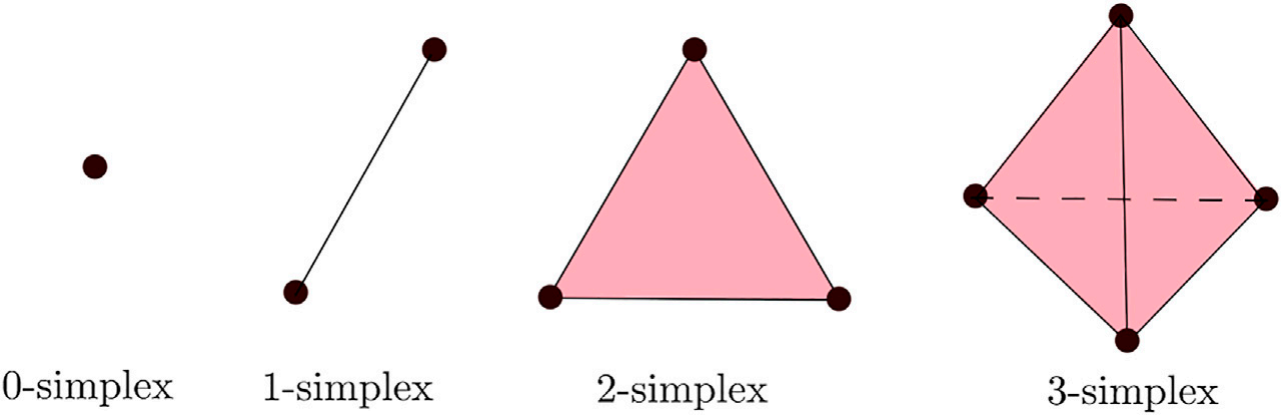
Simplexes of different dimensions.

We are interested in extracting the geometrical and topological features of the trajectories in D across different scales. For that purpose, we introduce the so-called *Rips filtration*. We construct a *Rips complex* from simplexes of varying dimensions that are generalizations of triangles of varying dimensions. More specifically, a *d*-simplex is the smallest convex set of *d* + 1 points, *x*_0_, … , *x*_*d*_ where *x*_1_−*x*_0_, … , *x*_*d*_−*x*_0_ are linearly independent, as illustrated in Figure 4. The so-called *abstract simplicial complex* is a finite collection of sets that is closed under the subset relation, i.e., if *a* ∈ *A* and *b* ⊂ *a*, then *b* ∈ *A*.

Let S be a trajectory, defined from a finite compact set of points in $R^{2}$ , and *ϵ* ≥ 0. The Rips complex of S at scale *ϵ*, $R_{\epsilon }\left(S\right)$ , is the abstract simplicial complex consisting of all subsets of diameter up to *ϵ*:

$$R_{\epsilon }\left(S\right)≔\left\{\sigma \subset S\;|\;\text{diam}\left(\sigma \right)\leq \epsilon \right\},$$

where the diameter of a set of points is the maximum distance between any two points in the set.

**FIGURE 5:**
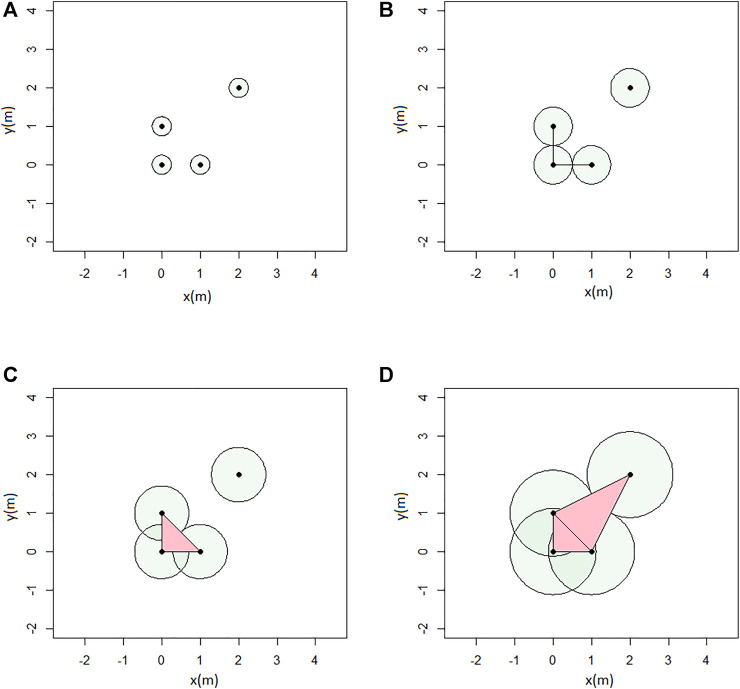
Example of Rips complex computation: **(A)** *ϵ* = 0.5; **(B)** *ϵ* = 1; **(C)** *ϵ* = 1.4; and **(D)** *ϵ* = 2.3 (units in meters).

Geometrically, we can construct the Rips complex by considering balls of radius $\frac{\epsilon }{2}$ , centred at each point in S . Whenever *d* balls have pairwise intersections, we add a *d*−1 dimensional simplex. An example of Rips complex is given in Figure 5.

A *filtration* of a simplicial complex K is a nested sequence of subcomplexes starting at the empty set and ending with the full simplicial complex

$$\emptyset \subset K_{0}\subset \cdots \subset K.$$

By varying the value of the scale parameter *ϵ*, from *ϵ*_min_ = 0 to $\epsilon_{\text{max}}=\text{diam}\left(S\right)$ we get a family of nested Rips complexes known as the Rips filtration.

### 2.3 Persistent Homology

In order to have a more exhaustive view on how the features are changing across different scales, the appearance and disappearance of each feature within the filtration is tracked and coded into the homology groups $H_{k}\left(S\right)$ , where *k* is the homology dimension. The elements of a *Homology Group* $H_{k}\left(S\right)$ are classes of chain of simplexes (“packets”) in the Rips complex. The use of homology groups allows us to perform algebraic operations over the simplicial elements. The homology group $H_{0}\left(S\right)$ represents the vertices, while the homology group $H_{1}\left(S\right)$ represents the cycles (loops) formed in the simplicial complex. Since our data is in $R^{2}$ we are only interested in *k* = 0 and *k* = 1.

Given a homology group, we can now define how to track the appearance of the features across different scales, by defining the homology group at a scale *ϵ*, $H_{k}^{\epsilon }\left(S\right)$ . It represents the classes of simplexes as described previously, but taken from $R_{\epsilon }\left(S\right)$ . That is, the elements of $R_{\epsilon }\left(S\right)$ with a filtration value lower than *ϵ*. This approach is known as the *persistent homology*. It allows to quantify the appearance and disappearance of the features across the different scales (discretized by considering *m* values related to *ϵ*_*j*_, *j* = 0, … , *m*):

• For $H_{0}\left(S\right)$ , the birth scale of all vertices is set to zero, while the death scale is the filtration value at which the vertex has been joined to another one by a segment.

• For $H_{1}\left(S\right)$ , the birth scale of a cycle is the filtration value at which a loop has been formed, while the death scale is the filtration value at which the interior of the loop has been covered.

We can formalize this as follows:

• The birth scale *b*_*γ*_ of the feature *γ*

$$b_{\gamma }=\underset{0\leq j\leq m}{\min}\left\{\epsilon_{j}:\gamma \in H_{k}^{\epsilon_{j}}\right\}$$

• The death scale *d*_*γ*_ of the feature *γ*

$$d_{\gamma }=\underset{0\leq j\leq m}{\max}\left\{\epsilon_{j}:\gamma \in H_{k}^{\epsilon_{j}}\right\}$$

The persistence of the features throughout the scales can then be represented by the so-called *persistence barcode* of S . It is a histogram, where the bar associated to each feature starts at the birth scale and ends at the death scale.

**FIGURE 6:**
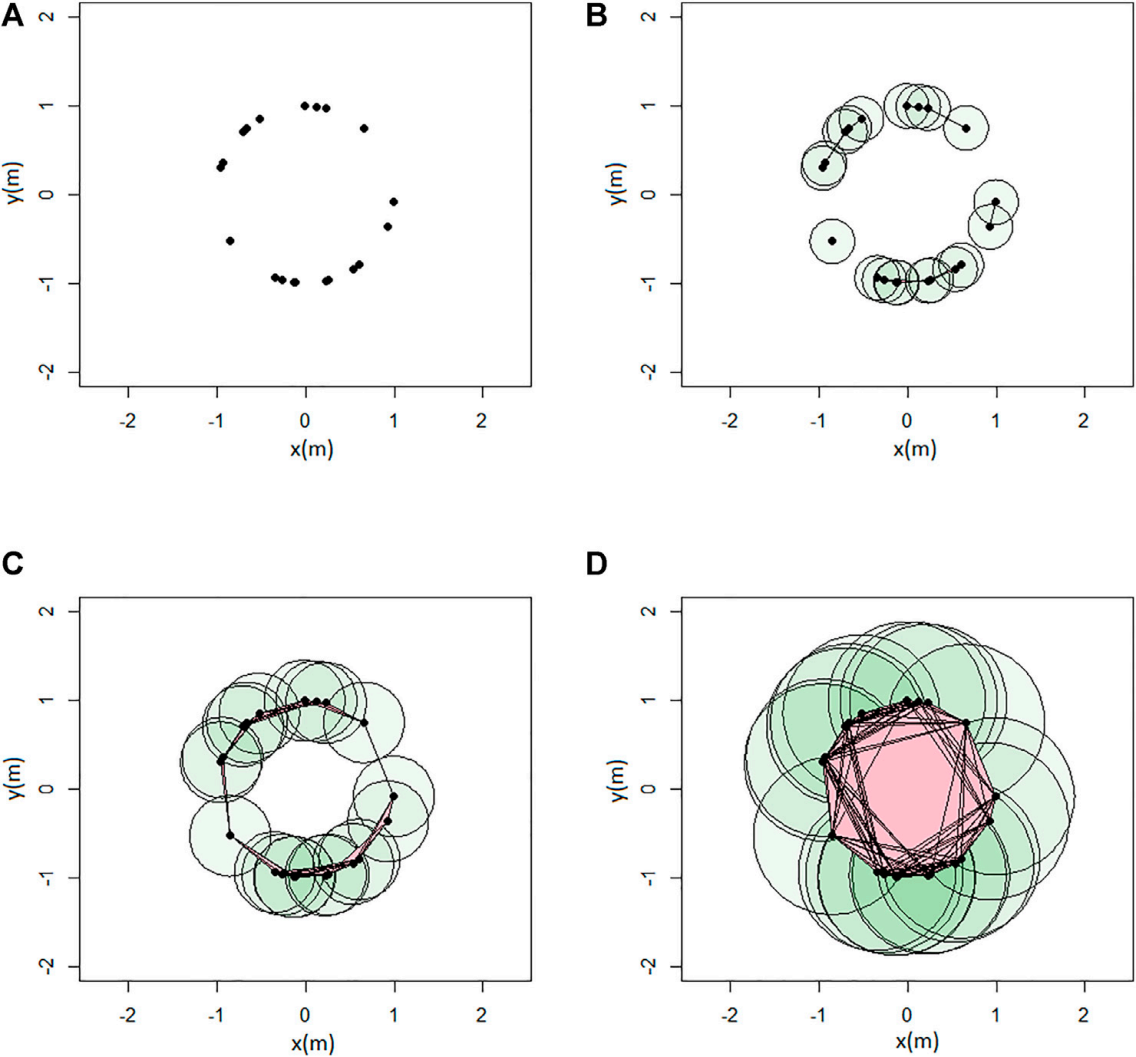
Example of Rips complex computation: **(A)** *ϵ* = 0; **(B)** *ϵ* = 0.5; **(C)** *ϵ* = 0.9; and **(D)** *ϵ* = 1.8 (units in meters).

**FIGURE 7:**
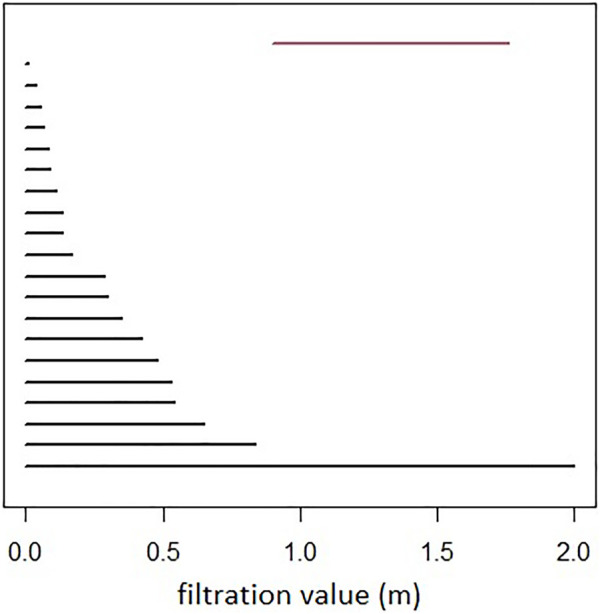
Persistence barcode: in black the *H*_0_ features, and in red the *H*_1_ feature. Filtration value (scale) is represented in the *x*-axis (units in meters).

An example of persistent homology computation is given with the rips complex in Figure 6, and the associated barcode in Figure 7. A loop is formed at *ϵ* = 0.9 (birth) and then covered at *ϵ* = 1.8 (death). It is represented by the red bar.

A more compact representation of the features persistence is the persistence diagram of S , defined from

$$\mathrm{PD}\left(S\right)=\left\{\left(b_{\gamma },d_{\gamma }\right):\gamma \in H_{k}\right\},$$

where *b*_*γ*_ and *d*_*γ*_ are the birth and death scales associated to the feature *γ*. In what follows, in the trajectories analysis, we only consider one-dimensional features, i.e., *k* = 1.

**FIGURE 8:**
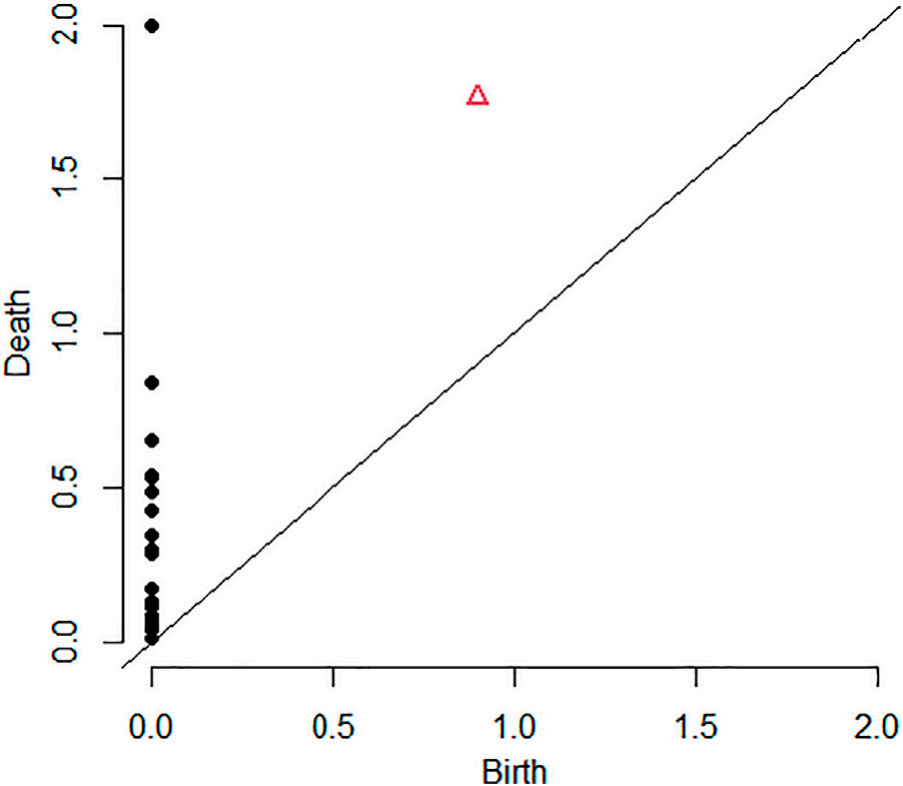
Persistence Diagram: in black the *H*_0_ features, and in red the *H*_1_ feature.

The persistence diagram associated with the Rips complex shown in Figure 6 is given in Figure 8. An equivalent representation of the persistence diagram consists in the so-called life-time diagram of S , which is constructed by means of a bijective transformation *T* (*a*, *b*) = (*a*, *b*−*a*), acting over PD(S) , that is,

$$\mathrm{LT}\left(S\right)≔\left\{\left(a,b-a\right)\in R^{2}:\left(a,b\right)\in \mathrm{PD}\left(S\right)\right\}.$$

In order to use the persistence features in a machine learning approach, we construct the so-called *persistent image* of S . First, observe that LT(S) is a finite set of *p* points,

$$\mathrm{LT}\left(S\right)=\left\{\left(a_{1},b_{1}-a_{1}\right),\ldots ,\left(a_{p},b_{p}-a_{p}\right)\right\},$$

and such that *b*_1_−*a*_1_ ≤ *b*_2_−*a*_2_ ≤ …, ≤ *b*_*p*_−*a*_*p*_. Then, consider a non-negative weighting function given by

$$\begin{array}{ll} w:\mathrm{LT}\left(S\right) & \to \left[0,1\right]\\ \left(a_{i},b_{i}-a_{i}\right) & \mapsto w\left(a_{i},b_{i}-a_{i}\right)=\frac{b_{i}-a_{i}}{b_{p}-a_{p}},\;\text{for}\;1\leq i\leq p. \end{array}$$

Finally, we fix *M*, a natural number, and take a bivariate normal distribution *g*_*u*_ (*x*, *y*) centered at each point u∈LT(S) with a variance $\sigma I_{2}=\frac{b_{p}-a_{p}}{M}I_{2}\;\left(I_{2}\right.$ is the 2 × 2 identity matrix). A persistence kernel is then defined according to:

$$\begin{array}{ll} \rho_{S}:R^{2} & \to R\\ \left(x,y\right) & \mapsto \rho_{S}\left(x,y\right)=\underset{u\in \mathrm{LT}\left(S\right)}{\sum }w\left(u\right)g_{u}\left(x,y\right). \end{array}$$

We associate to a robot trajectory $S\in R^{2}$ a matrix in $R^{M\times M}$ as follows: let *δ* > 0 be a non-negative, small enough real number, and then consider a squared region $\Omega_{S,\delta }=\left[a,b\right]\times \left[c,d\right]\subset R^{2}$ , covering the support of $\rho_{S}\left(x,y\right)$ up to a certain precision *δ*, such that

$$\iint_{\Omega_{S,\delta }}\rho_{S}\left(x,y\right)dxdy\geq 1-\delta .$$

Then, we consider two uniform partitions of the intervals

$$a=p_{0}\leq p_{1}\leq \ldots ,\leq p_{M}=b\text{ and }c=q_{0}\leq q_{1}\leq \ldots ,\leq q_{M}=d.$$

Finally, we express $\Omega_{S,\delta }$ from

$$\Omega_{S,\delta }=\overset{M-1}{\underset{i=0}{⋃}}\overset{M-1}{\underset{j=0}{⋃}}\left[p_{i},p_{i+1}\right]\times \left[q_{j},q_{j+1}\right]=\overset{M-1}{\underset{i=0}{⋃}}\overset{M-1}{\underset{j=0}{⋃}}P_{ij}.$$

The persistence image of S associated with the partition $P=\left\{P_{ij}\right\}$ is then described by the $R^{M\times M}$ matrix with elements:

$$PI{\left(S,M,P,\delta \right)}_{ij}=\left(\iint_{P_{ij}}\rho_{S}\left(x,y\right)dxdy\right)\text{ for }0\leq i,j\leq \left(M-1\right).$$

**FIGURE 9:**
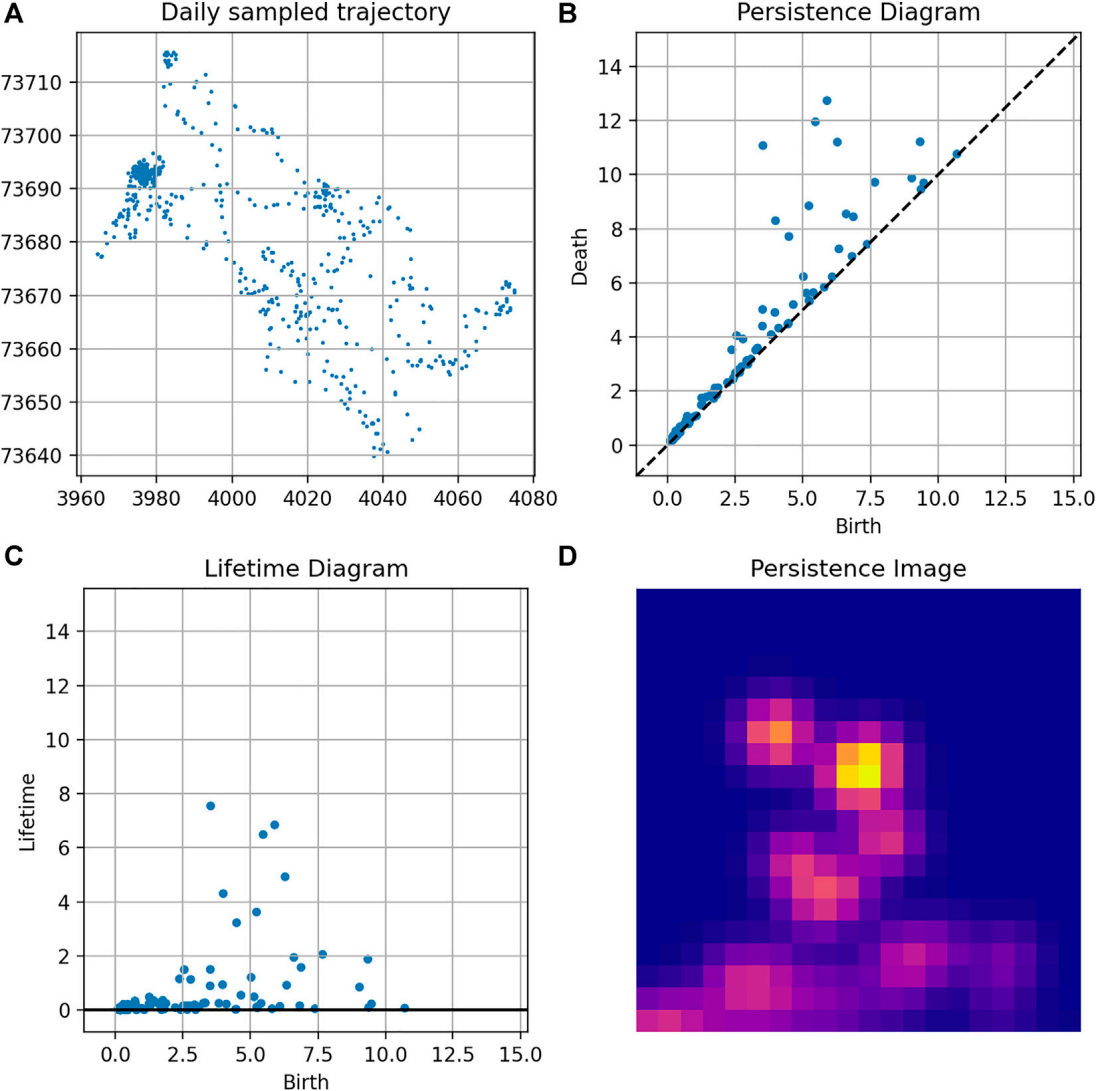
Topological analysis of a trajectory: **(A)** Trajectory; **(B)** Persistence diagram; **(C)** Lifetime diagram; and **(D)** Persistence Image.

An example of persistence computation for a given trajectory is given in Figure 9.

### 2.4 Measuring Persistence Similarity

Consider two data sets $S_{u}$ and $S_{v}$ representing two trajectories. A matching between two persistence diagrams, $\mathrm{PD}\left(S_{u}\right)$ and $\mathrm{PD}\left(S_{v}\right)$ , is a map *ψ*, that reads:

$$\psi :\mathrm{PD}\left(S_{u}\right)\to \;\mathrm{PD}\left(S_{v}\right),$$

such that $\forall \gamma =\left(b,d\right)\in \mathrm{PD}\left(S_{u}\right)$ ,

$$\begin{array}{ll} \psi \left(\gamma \right) & =\left(\psi_{1}\left(b\right),\psi_{2}\left(d\right)\right)\\ & =\left(b^{\prime },d^{\prime }\right)\in \mathrm{PD}\left(S_{v}\right). \end{array}$$

The map *ψ* associates each feature from $\mathrm{PD}\left(S_{u}\right)$ to a feature from $\mathrm{PD}\left(S_{v}\right)$ . The *optimal matching* between $\mathrm{PD}\left(S_{u}\right)$ and $\mathrm{PD}\left(S_{v}\right)$ is a matching $\hat{\psi }$

$$\hat{\psi }:\mathrm{PD}\left(S_{u}\right)\to \;\mathrm{PD}\left(S_{v}\right),$$

minimizing the transport cost C to move the features from $\mathrm{PD}\left(S_{u}\right)$ to $\mathrm{PD}\left(S_{v}\right)$ :

$$\begin{array}{ll} C_{\min}= & \underset{\gamma \in \mathrm{PD}\left(S_{u}\right)}{\sum }{\left\|\gamma -\hat{\psi }\left(\gamma \right)\right\|}_{2}\\ = & \underset{\left(b,d\right)\in \mathrm{PD}\left(S_{u}\right)}{\sum }{\left\|\left(b-{\hat{\psi }}_{1}\left(b\right),d-{\hat{\psi }}_{2}\left(d\right)\right)\right\|}_{2}\\ = & \underset{\left(b,d\right)\in {\mathrm{PD}}_{k}\left(S_{u}\right)}{\sum }\sqrt{{\left(b-{\hat{\psi }}_{1}\left(b\right)\right)}^{2}+{\left(d-{\hat{\psi }}_{2}\left(d\right)\right)}^{2}}. \end{array}$$

Then, to measure the degree of similarity between two trajectories $S_{u}$ and $S_{v}$ we consider the *Wasserstein distance* (Villani, 2006; Peyré and Cuturi, 2019) between $\mathrm{PD}\left(S_{u}\right)$ and $\mathrm{PD}\left(S_{v}\right)$

$$W\left(\mathrm{PD}\left(S_{u}\right),\mathrm{PD}\left(S_{v}\right)\right)=\underset{\left(b,d\right)\in \mathrm{PD}\left(S_{u}\right)}{\sum }\sqrt{{\left(b-{\hat{\psi }}_{1}\left(b\right)\right)}^{2}+{\left(d-{\hat{\psi }}_{2}\left(d\right)\right)}^{2}},$$

where $\hat{\psi }$ is the optimal matching between $\mathrm{PD}\left(S_{u}\right)$ and $\mathrm{PD}\left(S_{v}\right)$ .

**FIGURE 10:**
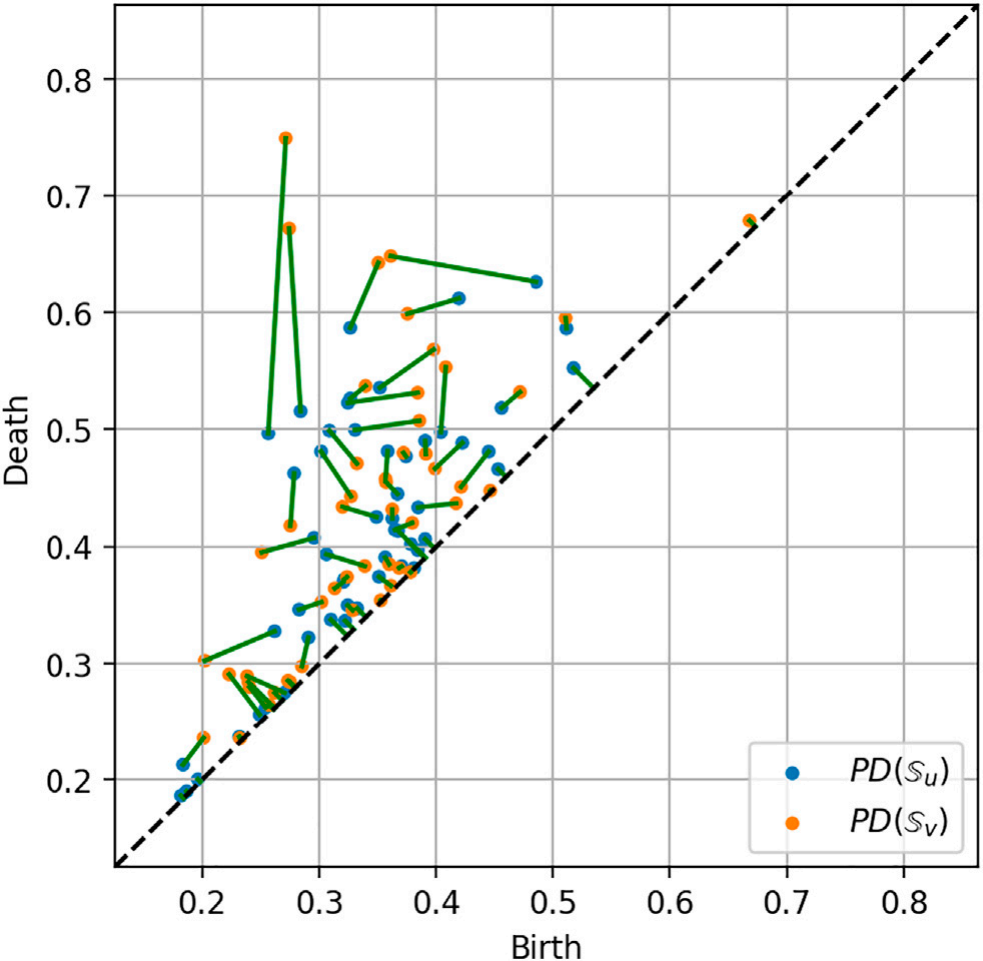
Optimal matching between two persistence diagrams related to two robot trajectories.

An example of matching between the persistence diagrams of two trajectories is given in Figure 10.

### 2.5 Barycentres of Persistence Diagrams

Consider now a collection $S_{1}\ldots S_{n}$ of trajectories with their associated diagrams ${\mathrm{PD}}_{1}\ldots {\mathrm{PD}}_{n}$ .

Since the space of persistence diagrams equipped with the Wasserstein distance, the *Wasserstein space*, is not a linear space, the notion of barycentres (Agueh and Carlier, 2011) can be extended for the persistence diagrams using the so-called *Frechet mean* (Turner et al., 2014), which always exists in the context of averaging finitely many diagrams.

The Frechet mean of ${\mathrm{PD}}_{1}\ldots {\mathrm{PD}}_{n}$ is any diagram minimizing the map

$$E:\mu \mapsto \overset{n}{\underset{i=1}{\sum }}W{\left(\mu ,{\mathrm{PD}}_{i}\right)}^{2}.$$

The computation of the barycentre *μ* has proven to be challenging, and multiple approaches can be used, such as the Sinkhorn algorithm (Cuturi and Doucet, 2014). We will use the one based on the Hungarian algorithm presented in Turner et al. (2014) and consider Partial Optimal Matchings (Divol and Lacombe, 2020), as the diagrams may not be of the same size. In this case, points from the diagonal are matched with the remaining (exceeding) points.

In our case, we estimate the barycentres of a finite family of persistence diagrams, taking a Lagrangian approach by tracking the individual points of the diagrams. Given a collection ${\mathrm{PD}}_{1}\ldots {\mathrm{PD}}_{n}$ of persistence diagrams, we proceed as follows:

1) Initialize the estimation *μ* of the barycenter at a certain diagram $\mu ={\mathrm{PD}}_{i_{0}}$ .
2) Compute the optimal partial matchings *ψ*_1_ … *ψ*_*n*_, between *μ* and ${\mathrm{PD}}_{1}\ldots {\mathrm{PD}}_{n}$ respectively.
3) Compute the updated barycentre $\hat{\mu }$ , by averaging the transport of each point in the barycentre *μ*

$$\hat{\mu }=\left\{y=\frac{1}{n}\overset{n}{\underset{i=1}{\sum }}\psi_{i}\left(x\right),x\in \mu \right\}.$$

4. If $\hat{\mu }$ minimizes E , return $\hat{\mu }$ . Otherwise, update $\mu =\hat{\mu }$ and go back to 2.

**FIGURE 11:**
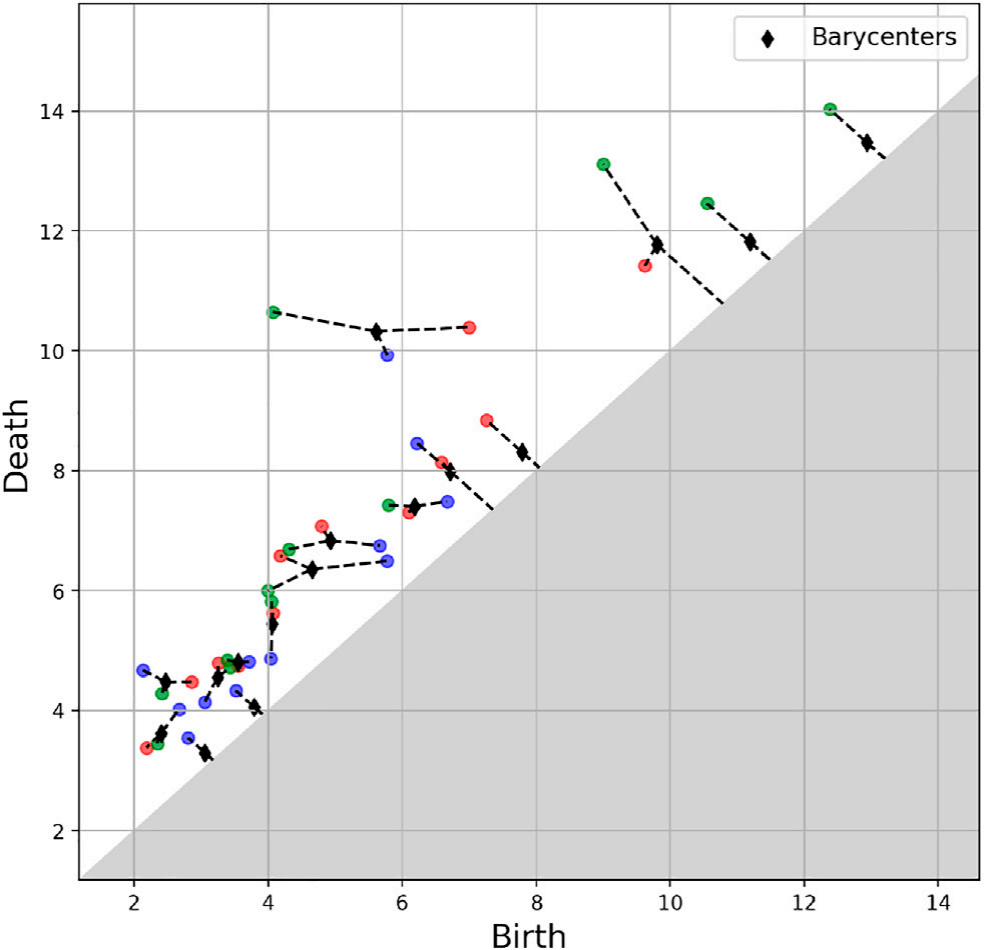
Barycentre (in black) of three persistence diagrams (red, blue and green).

An example of a barycentre of three persistence diagrams is given in Figure 11.

### 2.6 Classification

Image classification is a procedure that is used to automatically categorize images into classes by assigning to each image a label representative of its class. A supervised classification algorithm requires a training sample for each class, that is, a collection of data points whose class of interest is known. Labels are assigned to each class of interest. The classification problem applied to a new observation (data) is thus based on how close a new point is to each training sample. The Euclidean distance is the most common metrics used in low-dimensional datasets. The training samples are representative of the known classes of interest to the analyst. In order to classify the persistence images, we considered the logistic regression algorithm.

Consider a training set ${\left(X_{i}\right)}_{i=1}^{n}$ of flattened persistence images, i.e., *M* × *M*-component vectors, computed from a set ${\left(S_{i}\right)}_{i=1}^{n}$ of trajectories as described earlier. Associated is a list ${\left(Y_{i}\right)}_{i=1}^{n}$ of binary labels {0, 1}, describing whether an image $X_{i}$ is in the interest set or not.

The training of the $L_{2}$ *-penalized logistic regression binary classifier* is then the minimization of a cost function as described in the following optimization problem:

$$\min_{\omega ,c}\frac{1}{2}\omega^{T}\omega +C\overset{p}{\underset{i=0}{\sum }}\log\left(\exp\left(Y_{i}\left(X_{i}^{T}\omega +c\right)\right)+1\right).$$

Here *ω* are the weights we optimize over, *c* a Bernoulli mean vector of the weights, and *C* an inverse regularization parameter. Once trained, the model is evaluated on a unseen set of flattened persistence images. The metric used for the model evaluation is the *Accuracy Score* defined in the next section.

### 2.7 Model Evaluation

Evaluating a classification model consists of determining how often labels are correctly or wrongly predicted for the testing samples. In other words, it is counting how many times a sample is correctly or wrongly labelled into a particular class. We distinguish four qualities:

• TP (True Positive): the correct prediction of a sample into a class;
• TN (True Negative): the correct prediction of a sample out of a class;
• FP (False Positive): the incorrect prediction of a sample into a class;
• FN (False Negative): the incorrect prediction of a sample out of class.

These quantities are involved in the definition of the model performances estimator, the AccuracyScore (A). It is giver by the ratio of the number of correct predictions over the number of all samples, expressed by

$$A=\frac{TP+TN}{TP+TN+FP+FN}$$

## 3 Results

**FIGURE 12:**
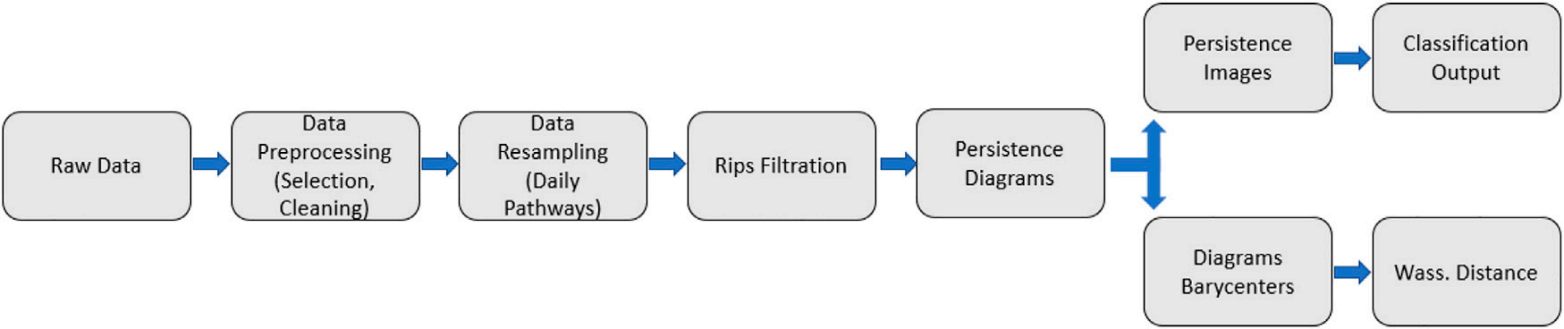
Workflow of the numerical procedure.

We recall the overall workflow of the proposed numerical procedure, summarized in Figure 12:

1) We start by preprocessing and cleaning the raw data gathered from the robot sensors
2) The data frequency is homogenized (minute data), and the pathways over each patch (or location) are daily sampled. We obtain 240 daily trajectories.
3) We compute the Rips filtration as described earlier, and then the persistence diagrams.
4) The persistence images are computed for each daily pathway, and used as inputs for the classification.
5) The persistence diagrams are also used to compute barycentres over given periods, and compute the Wasserstein distance between diagrams.

We also describe the two main classifications tasks at hand:

1) Predict the patch in which the robot is located every day, using the 240 daily persistence images as inputs. This is a binary classification task, associating to each daily persistent image either the label 1 if the robot is in the target patch, or the label 0 if the robot is in any other patch. The goal is to show the capacity to differentiate between pathways coming from different patches based on their topological signature.
2) Predict whether a maintenance has been operated on a robot or not, using the 50 daily persistence images associated to the same target patch as inputs. The maintenance dates are known. This is also a binary classification task, associating to each daily persistent image either the label 1 if the maintenance has occurred before that day, or the label 0 if not. The goal is to show the capacity to differentiate between functioning states of the robots, before and after a maintenance operation, while controlling the other factors (pathways sampled from the same patch).

The choice of the target patch #3 and has been motivated by two considerations:

• Have two equilibrated classes in the first classification task: the patch #3 is by far the one where the robot has spent most of time in operation, and it involves a very equilibrated 126/114 distribution of the two classes (0 and 1) after data cleaning.
• Also within the same patch it was possible to have 50 days time window around a maintenance operation where the robot stayed within the patch, with 25 days before and 25 after the maintenance operation on the robot, resulting in two perfectly balanced classes for the classification.
• We also note that the train-test split (65–35%) in both tasks has been done with stratification: the proportion of each class in the dataset is preserved when splitting the data (roughly 50–50%).

**FIGURE 13:**
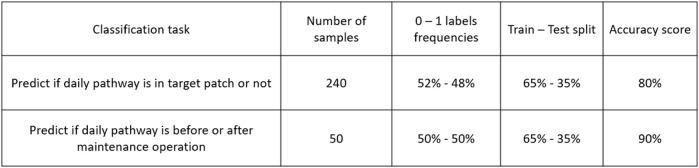
Classification tasks summary.

Both classification tasks and associated results are summarized in Figure 13.

### 3.1 Determination of the Patch in Which the Robot Is Located

We first want to predict whether a robot is in a certain patch. For that purpose we choose one parcel as a target, and train a classification model as described in Section 2.6. The complete dataset consists of daily trajectories for 240 days. For each day a persistence image is computed, which will then be used as input for the model (a sample is depicted in Figure 9). The samples are labelled according to the target patch (patch #3): 1 if the robot is in the target patch (114 samples), and 0 otherwise (126 samples). The dataset is split into 65*%* for training and 35*%* for testing. The proposed classifier achieves an 80*%* accuracy score in predicting the patch at which the robot is, based on the persistence images.

### 3.2 Maintenance Prediction

Then, we consider daily trajectories in the same patch (patch #3), consisting of 50 samples. For each day, a persistence image is computed, that will be used as input in the classifier. The periods considered here are the ones in between two consecutive maintenance operations of the robot. The samples are labelled 0 if they are associated to a day before the maintenance date (25 samples), 1 otherwise (25 samples). The dataset is split into 65*%* for training and 35*%* for testing. The model achieves a 90*%* accuracy score predicting the period associated to the sampled trajectories. The model high accuracy proves that the topological descriptors have enough information about the pathways to allow detecting patterns related to maintenance events, fact that could be used for predictive maintenance purposes.

### 3.3 A Time Varying Measure

**FIGURE 14:**
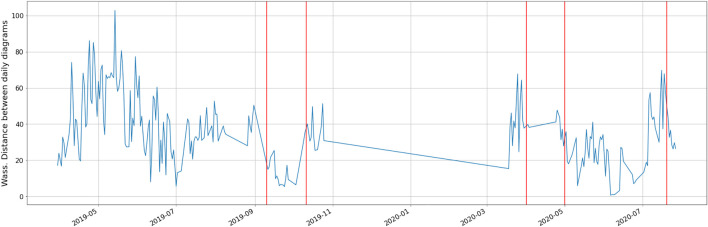
Time series of the Wasserstein distance between the persistence diagrams for consecutive daily trajectories: in red the maintenance events.

**FIGURE 15:**
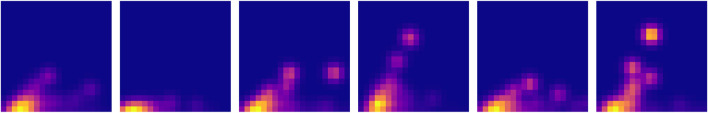
Persistence images of the barycentres computed for each period.

Figure 14 depicts the Wasserstein distance between the persistence diagrams for consecutive daily trajectories, with the maintenance operation emphasized in red, whereas Figure 15 shows the barycentres of each period between consecutive maintenance operations. As it can be noticed from the persistence images in Figure 15, maintenance operations affect the topology of the trajectory, as it was expected from the fact that classification performs successfully as just reported.

**FIGURE 16:**
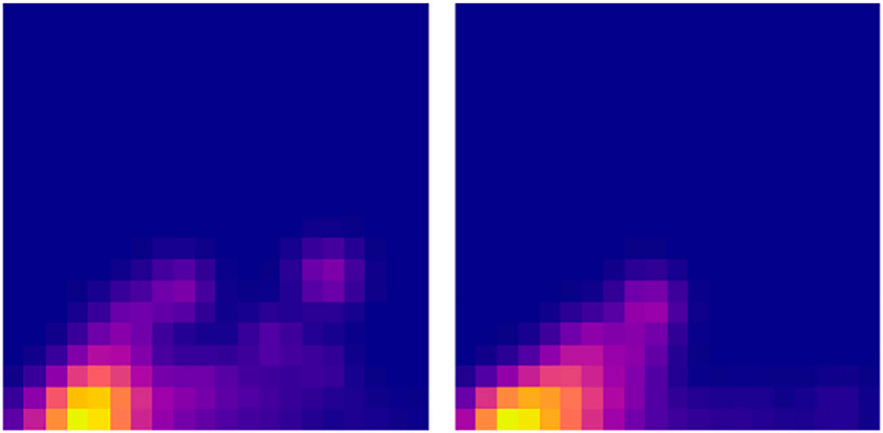
Persistence images of the two half-intervals related to the first period whose persistence image was the first image in Figure 15.

To better support our hypothesis about the effect of maintenance on the trajectory topology, we consider the first operation interval, the one before the first maintenance, that correspond to the first persistence image in Figure 15 (left), and divide it in two parts with identical length. Then, the associated barycentres in both half intervals are obtained. Both are represented in Figure 16.

As it can be noticed, both of them resemble very much to the one associated to the whole interval (the first picture in Figure 15), with a Wasserstein distance of 23.5 and 24.1 (computed on the associated diagrams). Conversely, the distance of both to the second period is much higher (46.5 and 26.1).

Finally, we can compute the distance of the 5 latter periods to the first one (Figure 15) and we have: 36.7, 31.0, 34.2, 33.3, 35.2, so significantly higher than the first period compared to its own two halves.

These results support again our assumption on the effect of maintenance on the trajectory topology.

## 4 Conclusion

The characterization of the trajectories followed by the robot based on the geographical location proves to be a reliable method to differentiate between different environments affecting the robot motion. Then, over a single patch, the classification was proved being efficient to detect the changes in the robot signature related to maintenance events.

The proposed topology-based framework for sampled trajectories seems a very pertinent, powerful and intrinsic way of quantifying, characterizing and analysing the topological and geometrical nature of the robot’s pathways. The strength of the framework relies on both the topology description of the trajectory at multiple scales, and the use of metrics features that can be combined with machine learning.

## Data Availability

The raw data supporting the conclusion of this article will be made available by the authors, without undue reservation.
